# Salicylic Acid- and Potassium-Enhanced Resilience of Quinoa (*Chenopodium quinoa* Willd.) against Salinity and Cadmium Stress through Mitigating Ionic and Oxidative Stress

**DOI:** 10.3390/plants12193450

**Published:** 2023-09-30

**Authors:** Sameera A. Alghamdi, Hesham F. Alharby, Ghulam Abbas, Habeeb M. Al-Solami, Afshan Younas, Majed Aldehri, Nadiyah M. Alabdallah, Yinglong Chen

**Affiliations:** 1 Department of Biological Sciences, Faculty of Science, King Abdulaziz University, Jeddah 21589, Saudi Arabia; saalghamdy1@kau.edu.sa (S.A.A.); hmalsolami@kau.edu.sa (H.M.A.-S.); 2 Plant Biology Research Group, Department of Biological Sciences, Faculty of Science, King Abdulaziz University, Jeddah 21589, Saudi Arabia; 3 Department of Environmental Sciences, COMSATS University Islamabad, Vehari Campus, Vehari 61100, Pakistan; afshanyounas007@gmail.com; 4 Department of Bio Sciences, COMSATS University Islamabad, Park Road, Islamabad 45550, Pakistan; 5 Anatomy Department, College of Medicine, King Khalid University, Abha 62217, Saudi Arabia; maldehri@yahoo.com; 6 Department of Biology, College of Science, Imam Abdulrahman Bin Faisal University, Dammam 31441, Saudi Arabia; nmalabdallah@iau.edu.sa; 7 Basic & Applied Scientific Research Centre, Imam Abdulrahman Bin Faisal University, Dammam 31441, Saudi Arabia; 8 The UWA Institute of Agriculture, UWA School of Agriculture and Environment, The University of Western Australia, Perth, WA 6001, Australia

**Keywords:** salicylic acid, potassium, cadmium stress, quinoa, oxidative stress, salinity

## Abstract

Salinity and cadmium (Cd) contamination of soil are serious environmental issues threatening food security. This study investigated the role of salicylic acid (SA) and potassium (K) in enhancing the resilience of quinoa against the combined stress of salinity and Cd. Quinoa plants were grown under NaCl (0, 200 mM) and Cd (0, 100 µM) stress, with the addition of 0.1 mM SA and 10 mM K, separately or in combination. The joint stress of Cd and NaCl caused >50% decrease in plant growth, chlorophyll contents, and stomatal conductance compared to the control plants. The higher accumulation of Na and Cd reduced the uptake of K in quinoa tissues. The joint stress of salinity and Cd caused an 11-fold increase in hydrogen peroxide and 13-fold increase in thiobarbituric acid reactive substances contents, and caused a 61% decrease in membrane stability. An external supply of 0.1 mM SA and 10 mM K helped plants to better adapt to salinity and Cd stress with less of a reduction in plant biomass (shoot 19% and root 24%) and less accumulation of Na and Cd in plant tissues. The activities of superoxide dismutase (SOD), catalase (CAT), peroxidase (POD), and ascorbate peroxidase (APX) were enhanced by 11-fold, 10-fold, 7.7-fold, and 7-fold, respectively, when SA and K were applied together to the plants subjected to the joint stress of Cd and salinity. Based on the values of the bioconcentration factor (>1), the translocation factor (<1), and the higher tolerance index, it was clear that Cd-contaminated, salty soils could be stabilized with quinoa under the combined supply of SA and K.

## 1. Introduction

Soil salinization is an increasing threat to sustainable crop production around the globe, including Pakistan [1]. The main damaging effects of salt stress on plants include an osmotic effect, ionic toxicity, and a reduction in photosynthesis, plant water uptake, gas exchange, and pigment contents [2,3,4]. For feeding the exponentially growing population of the world, the proper utilization of salt-affected soils by cultivating salt-tolerant plants such as quinoa (*Chenopodium quinoa* Willd.) seems a very promising option [1,5]. Quinoa is a halophyte plant and has less water requirements compared to other cereal crops including rice and wheat. Quinoa has a great nutritional value and its capacity to grow on compromised soils makes it a very promising crop for ensuring food security [1]. It has the potential to grow on contaminated saline soils, however, its growth is considerably decreased on metal-contaminated saline soils [6,7].

Soil contamination with cadmium (Cd) is a severe menace that has increased recently due to various human activities such as mining, agricultural practices, wastewater irrigation, and the discharge of industrial effluents [6,8]. Cadmium is ranked fourth among the toxic elements and its high solubility in water makes it particularly dangerous for plants [9,10]. It is a non-essential element because it does not have any biological function in plants [6]. When quinoa is grown under Cd contamination, its growth is severely declined due to disruption in its plant physiological attributes including a decline in the pigment contents and stomatal conductance of leaves [6,8]. The excessive accumulation of Cd in plant tissues results in the limited accumulation of essential plant nutrients such as potassium. Additionally, Cd-induced oxidative stress due to reactive oxygen species (ROS) results in the denaturing of many cellular organelles such as lipids, proteins, and nucleic acids [8,9]. The major ROS include hydrogen peroxide (H_2_O_2_), superoxide(O_2_^•−^), hydroxyl radicals (HO^•^), and singlet oxygen (½O_2_) [9]). Antioxidant enzymes, mainly superoxide dismutase (SOD), ascorbate peroxidase (APX), catalase (CAT), and peroxidase (POD), ameliorate the damage caused by ROS [1,8].

The exogenous supplementation of plants with different mineral nutrients and organic amendments has been found very promising for increasing the stress tolerance of plants [4,10]. Salicylic acid (SA) is an effective regulatory hormone against abiotic stresses [11]. Various mechanisms have been reported in plants under SA application to mitigate salinity stress such as increases in chlorophyll contents, leaf water contents, osmolytes accumulation, and the amelioration of oxidative stress by increasing the activities of antioxidant enzymes. Moreover, N, P, and K acquisition by plants is increased and Na accumulation is decreased [12,13]. The combined application of melatonin and SA increased salt tolerance in wheat by upregulating antioxidant machinery and the glyoxalase system [14]. SA helped the plants by compensating for a water deficiency and improved membrane stability [15]. It also ameliorated the phytotoxicity of Cd by regulating the antioxidant pathway and nutrient balance in mustard plants under Cd stress [11]. 

Potassium is an important macronutrient for the growth and metabolism of plants. Potassium has a well-established role for mitigating salinity stress in plants [4,16]. The external supply of K improved the water use efficiency and growth of quinoa exposed to salt stress [16]. Other possible mechanisms of K-induced salt tolerance include ionic homeostasis and the activation of antioxidant enzymes [4]. According to [17] Kausar and Gull (2014), K has a higher osmotic coefficient than Na and Cl, so under salt stress conditions, it is more capable of osmotic adjustment. Salt tolerant plants exhibit higher K and lower Na contents under salt-stress conditions [3,18]. Moreover, it has been reported that the exogenous K supplementation increases the resilience of plants against Cd stress. For example, [19] reported that in gladiolus (*Gladiolus grandiflora* L.), Cd stress was ameliorated due to the limited accumulation of metal ions by plants and enhanced activities of antioxidants. 

The individual role of K and SA under salinity and Cd stress has been well explored. However, their combined application for enhancing the tolerance of quinoa against the combined stress of salinity and Cd has not been investigated yet. It was hypothesized that the combined application of K and SA would be more effective than their sole application for enhancing the resilience of quinoa against the dual stress of Cd and salinity. This study aimed to explore the role of K and SA for increasing the resilience of quinoa against the combined stress of Cd and salinity, and to unravel the underlying mechanisms for the mitigation of ionic and oxidative stress caused by salinity and Cd. 

## 2. Results

### 2.1. Plant Growth

The results indicated that Cd and salinity stress considerably decreased the growth traits of quinoa. However, when both Cd (100 µM) and salinity (200 mM) were applied simultaneously, a higher decrease in the plant growth of quinoa was observed compared to Cd and salinity applied alone (Table 1). Under the combination of Cd and salinity, shoot and root lengths decreased by 60% and 57%, respectively, compared to the control. The exogenous treatments of 0.1 mM SA and 10 mM K significantly reduced the combined stress of Cd and salinity in quinoa plants. Compared to the joint treatment of salinity and Cd, the decrease in the shoot and root lengths was 42% and 47%, respectively, with the addition of 0.1 mM SA. Under the supply of 10 mM K, the reduction in the shoot and root length in plants facing the dual stress of Cd and salinity was 33% and 26%, respectively. However, the combined supply of SA and K was the utmost effective treatment combination for ameliorating the combined stress of salinity and Cd. The reduction in shoot and root lengths were 21% and 15%, when 0.1 mM SA and 10 mM K were applied to the plants under the joint treatment of salinity and Cd. The reduction in the shoot and root dry weight was 59% and 57%, respectively, under the combination of salinity and Cd with respect to control. Under the combined treatment of salinity and Cd, the decrease in these attributes was 38% and 51%, under the supply of SA. When K was applied to the plants under the joined treatment of Cd and salinity, the reductions in the shoot and root dry weight was 28% and 35%. When SA and K were applied together, the dry weight of the shoot and root declined by 19% and 24%, respectively, compared to the dual stress of Cd and salinity. 

### 2.2. Stomatal Conductance and Pigment Contents 

The combined treatment of Cd and salinity caused 50%, 59%, 52%, and 70% decreases in the pigment contents (chl a, chl b, total chl) and stomatal conductance, respectively, in comparison to the control (Table 2). The pigment contents (chl a, chl b, total chl) and stomatal conductance decreased by 39%, 39%, 40%, and 55%, respectively, when SA was applied in comparison to the plants facing the joint stress of Cd and salinity. Under the supplementation of K, the reduction in pigment contents (chl a, chl b, total chl) and stomatal conductance was 32%, 32%, 32%, and 40%, respectively, in comparison to the plants exposed to salinity and Cd together. Under the combined supplementation of SA and K for quinoa under the joint stress of Cd and salinity, the decrease in pigment contents (chl a, chl b, total chl), and stomatal conductance was 13%, 19, 11%, and 18%, respectively. 

### 2.3. Oxidative Stress Attributes 

**Figure 1:**
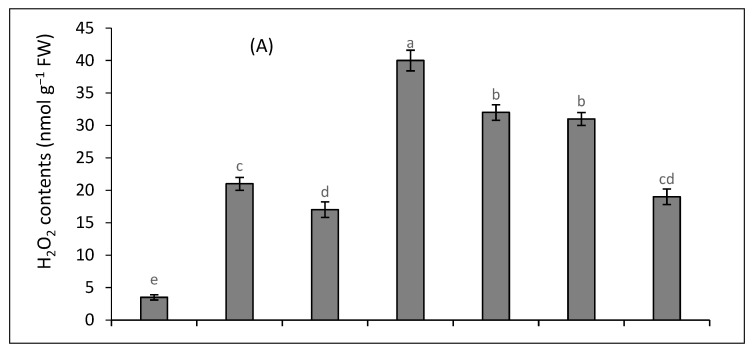

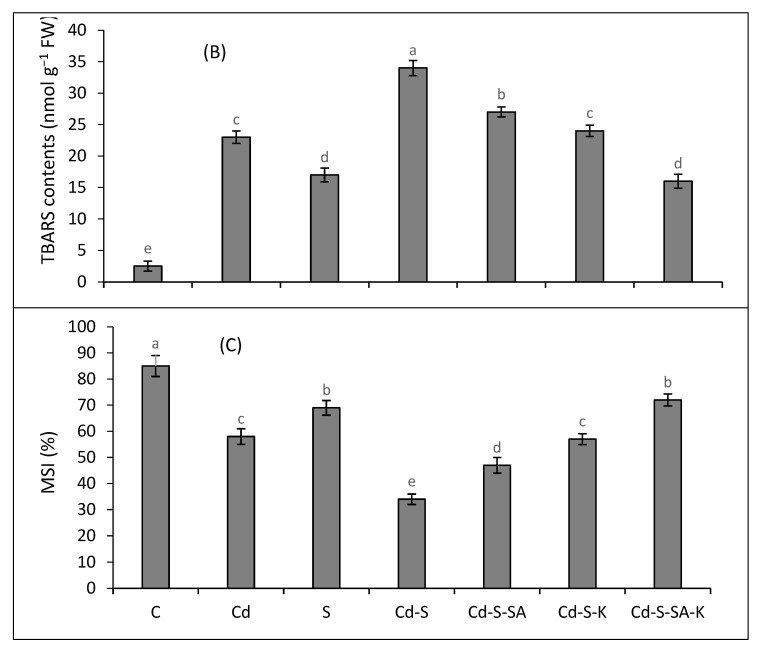
The impact of SA and K on H_2_O_2_ levels (**A**), (TBARS) levels (**B**), and MSI (**C**) of quinoa exposed to salinity (S), cadmium (Cd), and their combination. Distinct letters within every bar indicate a significant difference at a 5% level of significance.

The joint stress of Cd and salinity caused a greater production of H_2_O_2_ and TBARS than either stress alone (Figure 1A–C). Salinity and Cd treatments together increased the production of H_2_O_2_ (13-fold) and TBARS (11-fold) compared to the control. The MSI was decreased by 61% under the joint stress of salinity and Cd. The oxidative stress caused by Cd and salinity was decreased by the external supply of 0.1 mM SA and 10 mM K. The increase in the contents of H_2_O_2_ and TBARS was 9-fold and 10-fold in the presence of SA. The decline in MSI was 45% under the joint treatment of salinity and Cd under the supply of SA. Under the supply of K, the increase in the H_2_O_2_ and TABRS contents was eight-fold and nine-fold in plants growing under the joint stress of salinity and Cd. The decrease in MSI was 33% under the joint stress of salinity and Cd amended with K. The contents of H_2_O_2_ and TBARS were only five-fold and six-fold higher, and the reduction in MSI was 15 % when SA and K were applied together to the combined treatment of Cd and salinity.

### 2.4. Antioxidant Enzymes 

**Figure 2:**
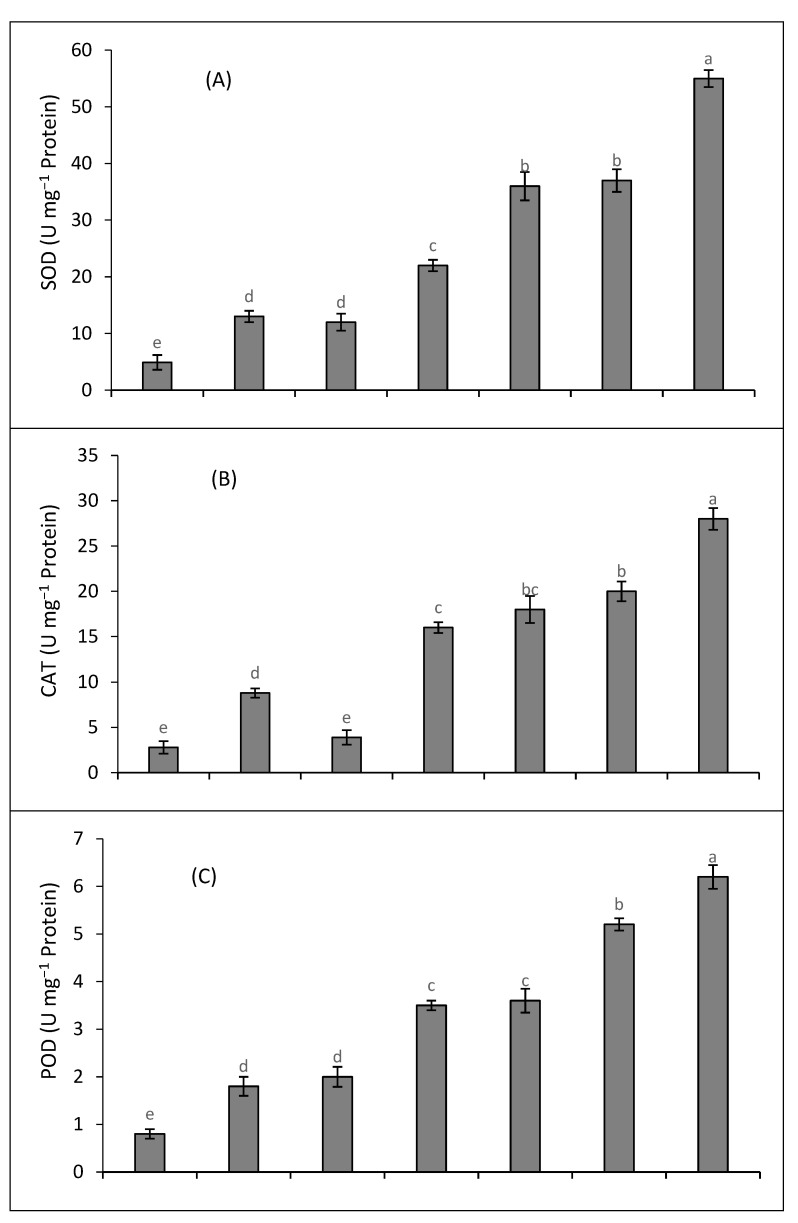

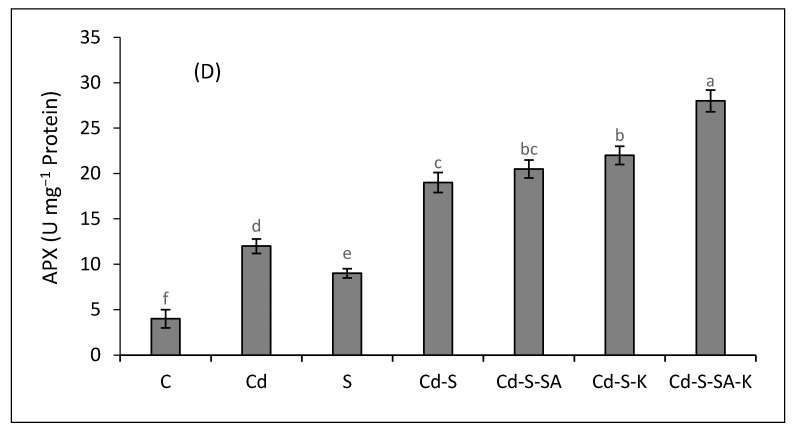
Impact of SA and K on the activities of SOD (**A**), CAT (**B**), POD (**C**), and APX (**D**) of quinoa plants under salinity (S), cadmium (Cd), and their combination. Distinct letters within every bar indicate a significant difference at a 5% level of significance.

Cadmium and salinity treatments resulted in the enhanced production of enzymes, e.g., SOD, CAT, POD, and APX (Figure 2A–D), in quinoa plants. The activity of these enzymes was further enhanced under the combined treatment of Cd and salinity. The results revealed that SOD, CAT, POD, and APX activities were enhanced by 4.5-fold, 5.7-fold, 4.3-fold, and 4.7-fold when the plants were subjected to the joint stress of salinity and Cd, with respect to the control. The exogenous application of SA and K caused a further increase in enzymatic activities. The activities of SOD, CAT, POD, and APX were increased by 7-fold, 6.4-fold, 4.5-fold, and 5-fold, respectively, under the joint treatment of Cd and salinity supplemented with SA. Likewise, K supplementation substantially increased the activities of these enzymes under the combined stress of Cd and salinity. The activities of SOD, CAT, POD, and APX were enhanced by 11-fold, 10-fold, 7.7-fold, and 7-fold, respectively, when SA and K were applied together to the plants subjected to the joint stress of Cd and salinity.

### 2.5. Metal Accumulation and Translocation

**Figure 3:**
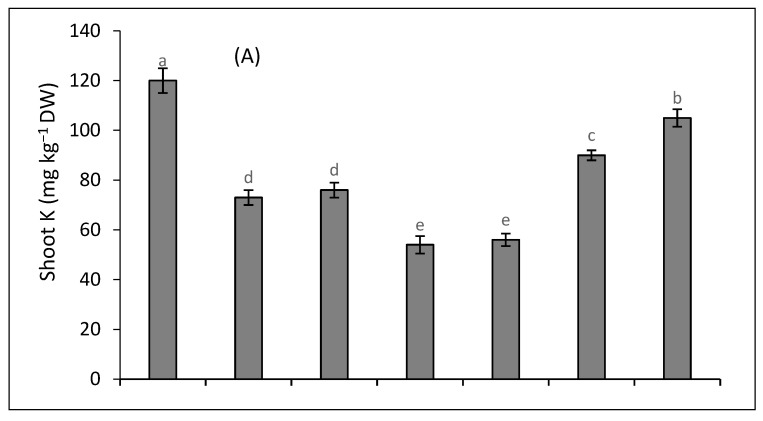

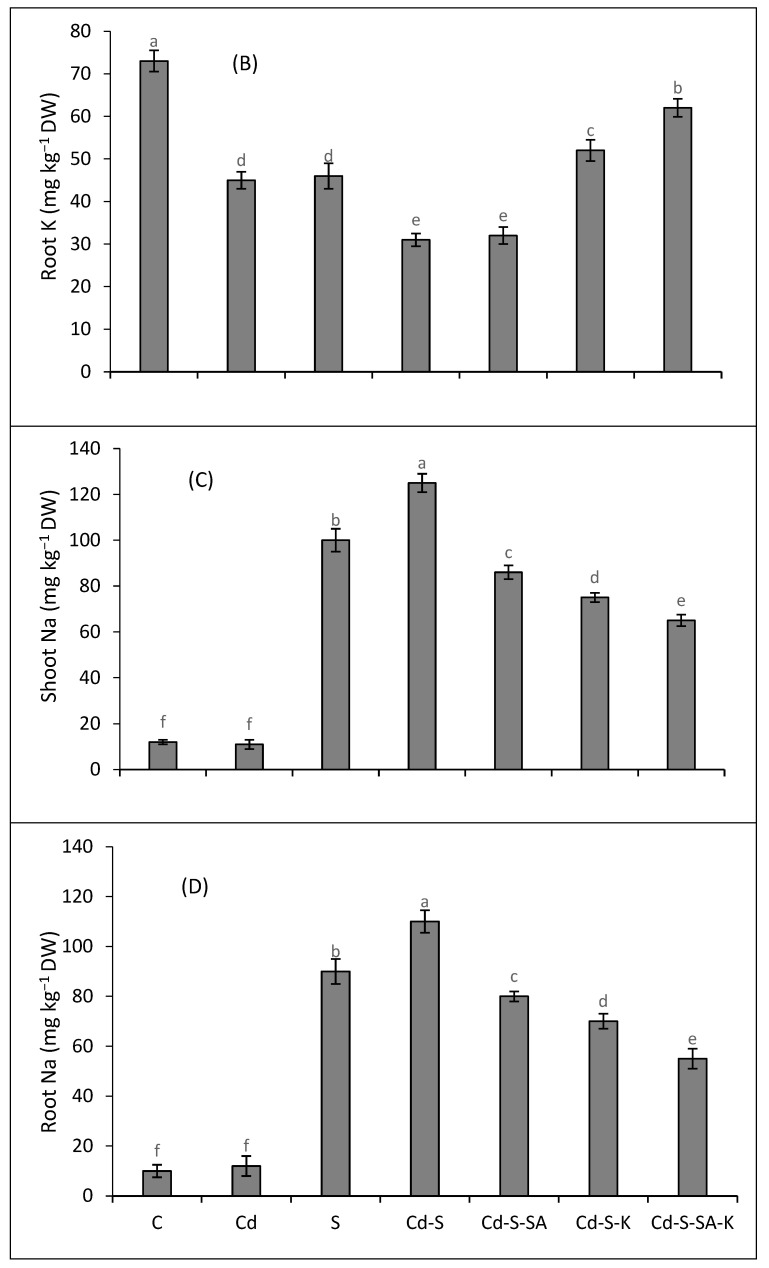
Impact of SA and K on shoot K (**A**), root K (**B**), shoot Na (**C**), and root Na (**D**) concentrations on dry weight basis of quinoa plants under salinity (S), cadmium (Cd), and their combination. Distinct letters within every bar indicate a significant difference at a 5% level of significance.

The accumulation of K in the roots and shoots of quinoa was decreased under salinity and Cd treatment compared to the control plants. However, K accumulation was further decreased when salinity and Cd treatment were applied together (Figure 3A,B). The root and shoot K contents were increased under the external supply of K and SA together. The accumulation of Na was increased under the joint stress of Cd and salinity (Figure 3C,D). The application of SA and/or K reduced the accumulation of Na both in the shoot and root of quinoa plants. The joint supply of SA and K further reduced the accumulation of Na in quinoa subjected to the combined stress of Cd and salinity.

**Figure 4:**
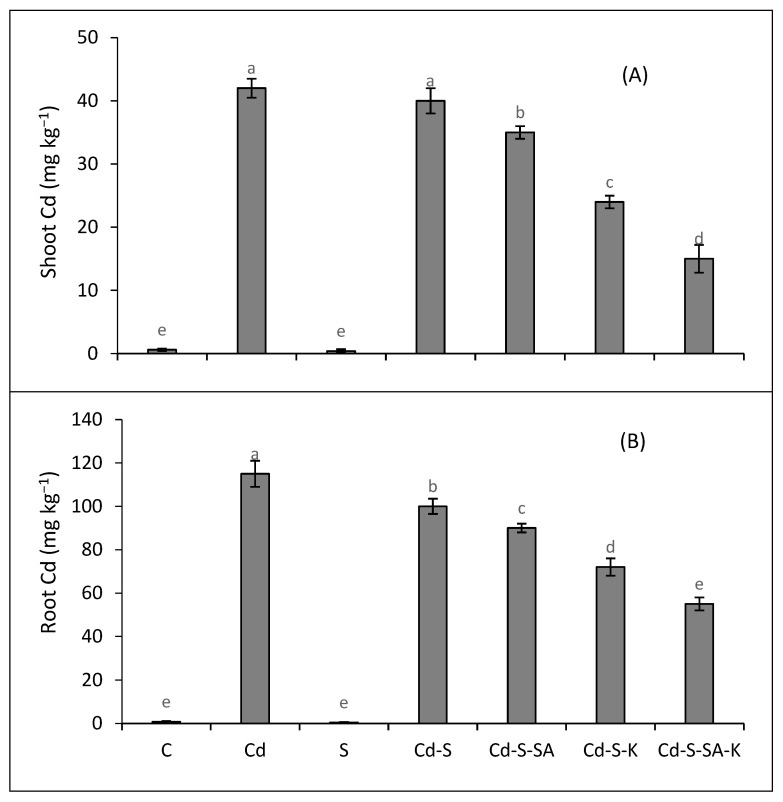
Effect of SA and K on shoot cadmium (**A**) and root cadmium (**B**) concentrations on dry weight basis of quinoa exposed to salinity (S), cadmium (Cd), and their combination. Distinct letters within every bar indicate a significant difference at a 5% level of significance.

The accumulation of Cd was higher in Cd-stressed plants with respect to the control. However, the joint stress of salinity and Cd further enhanced the accumulation of Cd in the shoot and root of quinoa (Figure 4A,B). The exogenous supply of SA and K, particularly in combination, restricted the accumulation of Cd in quinoa tissues. 

The values of BCF for Cd were higher than one, whereas the TF values were less than one for Cd alone or its combination with salinity (Table 3). A reduction in BCF and TF values was found under the exogenous supply of SA and K. However, the combination of SA and K resulted in the significantly lowest values of BCF and TF. As a result of the toxicity of salt and Cd, the TI of quinoa was decreased. The combined treatment of salinity and Cd resulted in the lowest TI. The application of SA and K, especially in combination, increased the TI of quinoa growing under salinity and Cd stress.

### 2.6. Multivariate Analyses

**Figure 5:**
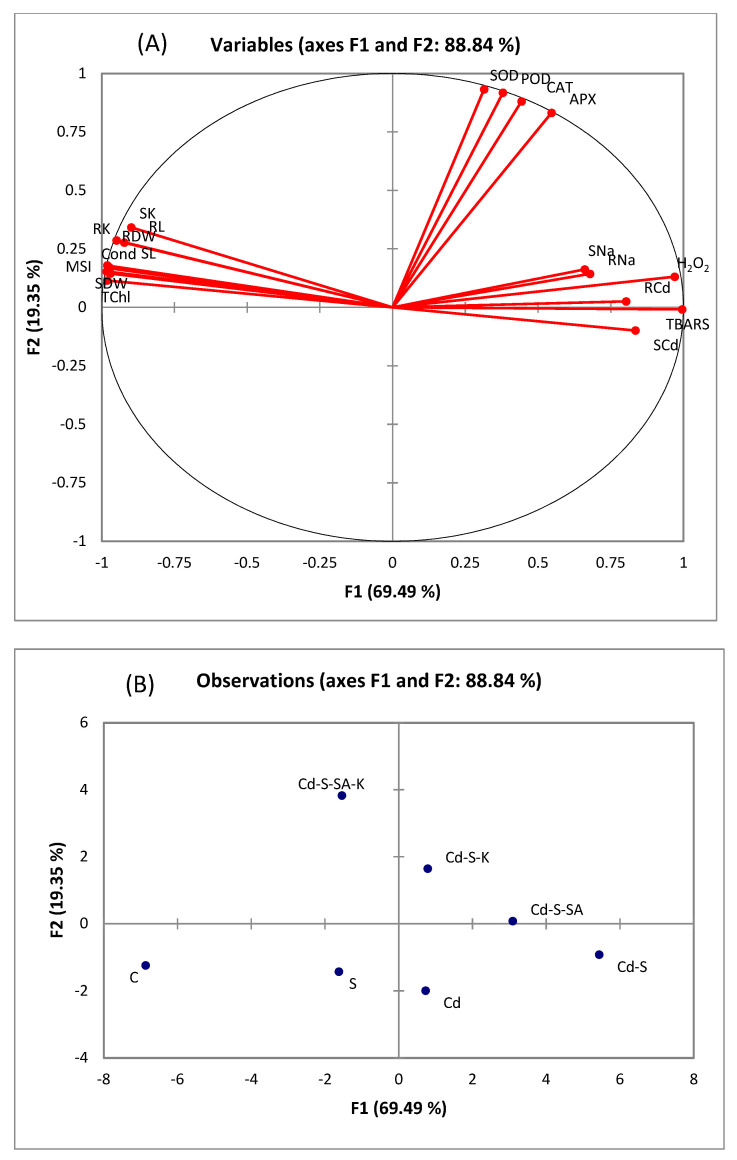
Principal component analysis of response variables of quinoa (**A**) and different treatments of salinity (S), cadmium (Cd), and their combination (**B**) with or without supplementation of SA and K.

The relationships amongst the variables in the data set were explored using Pearson correlations matrix and PCA, as shown in Figure 5 and Table 4. Although six factors contributed to the data variability, the main contribution to the total variability was only from three factors which contributed 69%, 19%, and 9%, respectively. As indicated in Figure 5A, the variables were clustered into three groups based on the close association: (1) plant growth-related attributes were placed together with physiological attributes, (2) antioxidant enzymes were placed closer to each other, and (3) the contents of Cd, Na, H_2_O_2_, and TBARS had close association. Different treatments were also grouped into various axes, as shown in Figure 5B. The left side of the F1 axis contained the control and salinity treatments which were categorized by relatively more growth, stomatal conductance, and pigments, as compared to Cd treatments. The right side of the F1 axis contained the combined treatment of salinity and Cd that was related to the higher levels of the Na and Cd contents, as well as elevated oxidative stress. The combined treatments of salinity and Cd either supplemented with K or SA treatments were found at the lower side of the F2 axis. The combined treatment of salinity and Cd along with K and SA together was found at the upper side of the F2 axis and was associated with better plant growth, and the lowest metal contents and oxidative stress.

The Pearson correlations matrix (Table 4) indicated the strong positive relationships of the K concentration in plant tissues with plant growth, stomatal conductance, and pigment contents. On the other hand, oxidative stress attributes have strong negative associations with the K contents in plants. Stomatal conductance and pigments correlated negatively with the root and shoot Cd contents, whereas oxidative stress attributes correlated positively with the Cd contents. 

## 3. Discussion

The current study was undertaken to explore the role of the exogenous supply of SA and K for the mitigation of salinity and Cd stress in quinoa plants. It was observed that both the salinity and Cd decreased plant growth attributes; however, their combined stress was more detrimental for the plant growth of quinoa. A decline in the growth of quinoa and other plants under Cd stress is well reported [6,20,21]. Cadmium, after being taken up by plant roots, is accumulated in different parts of the plants [22]. Cadmium and its compounds are included in the first-priority hazardous chemicals due to their harmful effects and carcinogenic nature [23]. The growth reduction of plants due to Cd is mainly attributed to the reduction in the chlorophyll and stomatal conductance of plants leaves, limited uptake of essential nutrients such as K, and oxidative stress caused by the over production of various ROS [6,24,25]. 

The growth of the quinoa plants was decreased under salt-stress conditions. It has been reported that plants facing salinity stress show different responsive mechanisms at a cellular homeostasis level [26]. A salinity-induced decline in plant growth is attributed to an osmotic effect, specific ion toxicity, and a decline in pigment contents, photosynthesis, gas exchange, and plant water uptake [2,3,4]. Moreover, the denaturing of macromolecules due to oxidative stress also is a major contribution in limiting plant growth under salinity stress [1,2]. The combined stress of Cd and salinity was particularly harmful for quinoa plants. A more severe decline in plant growth under the combined stress of Cd and salinity might be due to the disturbance in photosynthetic activities, damage to the cell membrane structure, and disruption in water balance [24]. Moreover, Cd and salt stress affected many essential processes of plants such as the over-production of ROS, decrease in stomatal conductance and pigment contents, and disturbance in the uptake of essential nutrients including K [6,21].

**Figure 6:**
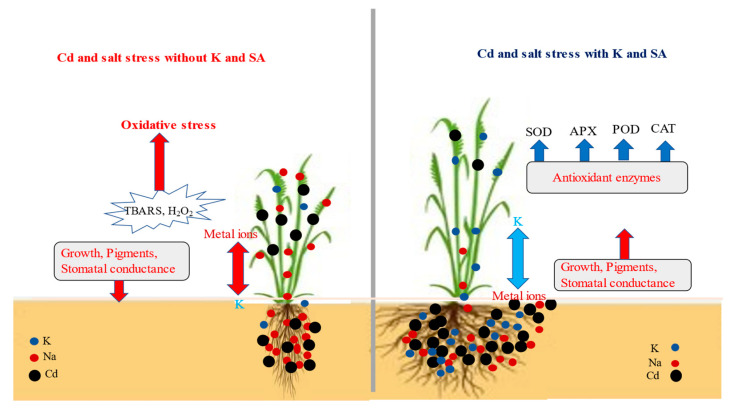
Effect of SA and K on plant growth, physiology, and oxidative stress responses of quinoa facing the dual stress of Cd and salinity. The combined application of SA and K improved plant physiological attributes and decreased the oxidative stress by limiting the accumulation of Cd and Na.

When quinoa was subjected to the exogenous supply of SA and K, particularly their joint application, its growth attributes were improved (Table 1; Figure 6). Such an ameliorative role of the foliar supply of SA for plants facing salinity and heavy metal stress has been reported in many earlier studies. For example, SA applied in foliar spray improved the growth attributes of *Iris pseudacorus* L. plants under saline stress [15], and of mustard plants facing Cd stress [11]. The mechanisms of SA-induced growth improvement include the stability of the cell membrane [27], the mitigation of ROS through the enhanced activities of antioxidant enzymes [28,29,30], the restricted accumulation of toxic ions, and increased stomatal conductance and photosynthetic activities [20,31]. 

Potassium is a macronutrient, and its exogenous supply is very beneficial for plants under normal and stressful conditions [4]. The exogenous supply of K decreased the toxic effects of Cd and salinity in quinoa, as noted for some other plants [4,32]. Likewise, [33] Alharby et al. (2022) also revealed that K supplementation combined with silicon enhanced the tolerance of quinoa against lead and Cd. The possible mechanisms of the K-induced improvement in plant growth under salinity and Cd stress may improve stomatal conductance, enhanced water and nutrient uptake, ionic homeostasis, and the activation of antioxidant enzymes to mitigate the oxidative stress [4,33].

In our study, the combined supply of K and SA was the most effective treatment for quinoa plants against the dual stress imposed by Cd and salinity, as indicated in Figure 6. Likewise, the application of SA and K combined with some other nutrients has been reported to mitigate salinity and Cd stress in various plants [11,19,34]. The possible reasons for better growth under the combined treatment of SA and K include enhanced water and nutrient uptake, stomatal conductance, photosynthetic activities, the restricted accumulation of toxic ions, and the activation of antioxidant enzymes [20,27,31].

In our study, the combined stress of Cd and salinity caused more damage to the stomatal conductance and pigment contents compared to either stress alone. Many other studies also reported the decrease in pigment contents and stomatal conductance in various plants including quinoa under salinity, Cd, and their combination [6,35,36,37,38]. A further decline in the stomatal conductance and pigment contents under the dual stress of Cd and salinity might be due to the direct toxicity of ROS or denaturing of the chlorophyll structure due to the replacement of magnesium by Cd or/and Na ions within chlorophyll molecules [21,39,40,41]. The exogenous supply of SA increased the pigments and stomatal conductance in quinoa plants. Likewise, ref. [29] also reported that SA enhanced the pigment contents and stomatal conductance in Brassica campestris plants under lead stress. It has been reported that SA binds to toxic metals and limits their accumulation and uptake by plants and increases the availability of essential nutrients such as Ca, Mg, and K for plants [20]. The external supply of K also increased the pigment contents and stomatal conductance in quinoa plants. Such positive effects of K have been observed in many other plants as well and are linked to improved physiological functions in the presence of K, a reduction in toxic ion (Cd and Na) accumulation, and the mitigation of ROS [33,36,42]. Under the combined treatment of SA and K, the above-mentioned effects were additive, hence, there was a greater increase in the pigments and stomatal conductance of plants than with the individual application of SA and K. 

Cadmium and salinity caused the overproduction of H_2_O_2_ and TBARS in quinoa plants, resulting in membrane instability. This oxidative stress was more detrimental under the joint stress of Cd and salinity. Many previous studies also reported the membrane damage and induced oxidative stress in many plants under the joint stress of Cd and salinity. For example, Abdal et al. [6] found that the combined stress of salinity and Cd caused membrane damage due to the overproduction of H_2_O_2_ in quinoa plants. Likewise, Khan et al. [43] observed that the combined treatment of salinity and Cd was more detrimental than their separate application in terms of oxidative damage for *Phoenix dactylifera* L. The mitigation of ROS in plants is carried out with the help of numerous antioxidant enzymes [13,35]. The most important antioxidant enzyme is the SOD because it converts superoxide radicals into H_2_O_2_ and oxygen [7,13]. Our results indicate that the activity of SOD was increased under the joint treatment of SA and K. The increased activity of SOD under the exogenous supply of SA and K has been noticed in many plants facing Cd and salinity stress [17,19,30,32]. The H_2_O_2_ is converted into water and oxygen by the other antioxidant enzymes including CAT, POD, and APX [4,33]. We found that the activities of these enzymes were particularly enhanced under the combined supply of SA and K. Therefore, it can be concluded that both SA and K combined alleviated the oxidative stress in quinoa plants by increasing antioxidant enzyme activities [20,29,32].

We found that the uptake of K was decreased due to excessive accumulation of Cd and Na both in the roots and shoots of quinoa [7,21]. The exogenous application of K combined with SA resulted in the enhanced uptake of K and reduced the accumulation of Cd and Na [32]. Potassium has a well-established role in the mitigation of salinity and Cd stress in plants [4,16,19,44]. The external supply of K improves water use efficiency, ionic homeostasis, and the activation of antioxidant enzymes, and limits the accumulation of metal ions by plants [4,16,19]. Our results are in line with previous studies which concluded that SA limits the accumulation of Na and Cd in different plants [12,13,45,46]. The lowest accumulation of Cd and Na in quinoa tissues was under the joint application of SA and K; hence, the combined application of SA and K was the most promising treatment for the mitigation of the combined stress of salinity and Cd in quinoa.

It was observed that the value of BCF for Cd was more than one, however, the value of TF was less than one. This type of metal uptake and translocation pattern in quinoa has been found in many previous studies [6,7,21]. Both BCF and TF for Cd were decreased under the sole application of SA and K; however, significantly lower values of both BCF and TF were noticed under the joint supply of SA and K. The decrease in TI of quinoa under Cd stress can be attributed to the phytotoxicity of Cd to plant growth attributes. However, the joint application of SA and K increased the TI of quinoa. This increase in TI was due to an increase in dry weight of plants due to the ameliorative effects of SA and K on plant physiological attributes. The values of BCF, TF, and TI showed that the quinoa genotype Puno can be cultivated on soils simultaneously contaminated with Cd and salinity. Additionally, the exogenous joint supplementation of SA and K should be done to increase the tolerance of quinoa plants against the combined stress of Cd and salinity. 

The PCA was used for the comparison of different response variables and treatments. This is an ideal way of data analysis to depict the relationships between different variables [1]. The PCA indicated that stomatal conductance, pigments, and growth attributes of plant were grouped together. The oxidative stress traits, and shoot and root Na and Cd contents were grouped together. The Pearson correlation matrix also confirmed this close association of the above-mentioned groups. Strong negative correlations were found among the plant growth and physiological features with the Cd contents in the roots and shoots of quinoa. Likewise, plant growth and physiological attributes showed significant negative associations with the oxidative stress responses of the plants. The K contents of roots and shoots revealed positive correlations with plant growth, stomatal conductance, and pigment contents, whereas strong negative correlations with oxidative stress attributes. These observations are in line with our results where K, along with SA, had improved plant growth and physiological attributes and decreased the contents of H_2_O_2_ and TBARS. The presence of different treatments in opposite axes was also in line with their effects on different attributes of quinoa. It was demonstrated that the combined treatment of salinity and Cd was more detrimental for quinoa than the sole treatments. The presence of the joint treatment of K and SA at the top of the y-axis indicated that their combined application had more beneficial effects than their separate application for quinoa facing the dual stress of salinity and Cd.

## 4. Materials and Methods

### 4.1. Growth Conditions and Treatments

The seeds of quinoa (*Chenopodium quinoa* Willd.) genotype Puno were taken from the Department of Environmental Sciences, COMSATS University Islamabad, Vehari Campus, Pakistan. Seedlings were raised in acid-washed sand and three-week-old seedlings were transferred to half-strength Hoagland’s nutrient solution. Plants were allowed to adjust for one week and then exposed to various levels of salinity (0, 200 mM) and Cd (0, 100 µM) alone or in combination (200 mM NaCl + 100 µM Cd). Sodium chloride (NaCl) salt was used for salinity treatment and cadmium chloride (CdCl_2_) was used for Cd treatment. NaCl was applied in two steps: half was applied with Cd treatments, while the other half was applied after two days of Cd treatment. Each time, 5.85 g of NaCl were dissolved per liter of solution, whereas 18.3 mg of CdCl_2_ were added once per liter of solution. One week after the treatments of salinity and Cd, plants were supplied with 10 mM K in solution and 0.1 mM SA as foliar application. Foliar application was continued for two weeks on alternate days. The solution was changed every week and dilute sodium hydroxide, or hydrochloric acid were used to maintain the pH of solution at 6.5 ± 0.2. This study had a completely randomized design (CRD), with four replications of each treatment. There were two plants of quinoa in each replication. 

### 4.2. Plant Growth Measurements

Harvesting of the plants was done after four weeks of exposure to the stresses, and growth data were recorded. Shoot and roots were harvested separately. Roots and shoots lengths were measured using a measuring tape. Root and shoot samples were air dried before oven drying for 48 h at 65 °C. Dry weight of roots and shoots was recorded separately using a digital weighing balance.

### 4.3. Metal Analyses 

Plant dry samples were digested in HNO_3_ and HClO_4_ in 2:1 ratio on a hot plate [47] (AOAC 1990). Filtration was done using Whatman filter paper No. 42, and samples were diluted in double-distilled water to a maximum of 50 mL volume. Potassium and Na concentrations were measured on a flame photometer (BWB-XP5), while Cd concentration was determined using atomic absorption spectrophotometer (PerkinElmer Model: PinAAcle900F Inc., Waltham, MA, USA). Single-element calibration standards (GBW 10016 in tea leaves) were used to ensure the accuracy of the results. 

### 4.4. Leaf Pigments and Stomatal Conductance

Fresh leaf samples were collected, and 1.0 g of each sample was ground using a mortar and pestle. Pigment contents (Chl a, Chl b, and total Chl) were extracted using a buffer solution of hydro-acetone (80% *v*/*v*). The resulting extracts were centrifuged at different wavelengths (663.2, 646.8, and 470 nm) for 10 min, using a UV-Vis spectrophotometer (Lambda 25, PerkinElmer, Inc., Los Angeles, CA, USA) in accordance with the method described by Lichtenthaler (1987) [48]. The pigment contents were expressed on fresh weight basis of the leaves. The second newly emerged leaf was used for measuring the stomatal conductance using a portable leaf porometer (Decagon Devices, Pullman, WA, USA) on a clear sunny day [49].

### 4.5. Oxidative Stress Attributes

Hydrogen peroxide (H_2_O_2_) was determined as described by [50] Islam et al. (2008). To homogenize 0.5 g of leaf samples, 0.1% trichloroacetic acid was utilized. After that, samples underwent a 20-min centrifugation at 12,000× *g*. The reaction mixture consisted of one mL supernatant, potassium iodide (1 mL, 2 M), and potassium phosphate buffer (1 mL, 10 mM) at pH at 7.0. A UV visible spectrophotometer was used to analyze this mixture at wavelength of 390 nm and the concentration of H_2_O_2_ in the sample was estimated. The lipid peroxidation was determined by quantifying the levels of thiobarbituric acid reactive substances, as described by [51] Hodges et al. (1999).

### 4.6. Membrane Stability Assay

Membrane stability was measured at two different temperatures, i.e., 40 and 100 °C, as described by [52] Sairam et al. (2002), by noticing the EC of leaf leachate in double distilled water. We took 0.1 g of leaf samples, chopped them into small and evenly sized disks, and put them into test tubes. Each test tube contained 10 mL distilled water in separate sets. Two test tube sets were prepared, one of which was kept at 40 °C for 30 min, and the other was kept at 100 °C for 15 min. The electric conductivities of each set (C1 and C2) were recorded using an EC meter. The following formula was used for calculating the MSI value.

MSI = 1 − (C_1_/C_2_) × 100

### 4.7. Enzymatic Activities

To measure the antioxidant enzymes, fresh and fully grown leaves of the quinoa plants were collected before the final harvest. Using a 0.1 M phosphate buffer solution at pH 7, the leaf samples (0.5 g) were ground. For 30 min, leaf samples were centrifuged at 15,000× *g* at 4 °C to obtain leaf extract. The activity SOD was evaluated using the method of Dhindsa et al. (1981) [53], in which the nitro blue tetrazolium concentration was reduced by 50%. The activity of APX was determined according to the method described by Nakano et al. (1981) [54] and expressed as 1 M ascorbate min^−1^ mg^−1^ protein. CAT activity was assayed using the procedure outlined by Aebi et al. (1984) [55] and expressed as µM of H_2_O_2_ decomposed min^−1^ mg^−1^ protein. The enzymatic activity of POD was measured using the method described by Hemeda et al. (1990) [56] and expressed as µM guaiacol oxidized min^−1^ mg^−1^ protein.

### 4.8. Bioconcentration Factor (BCF) and Translocation Factor (TF)

The bioconcentration factor (BCF) and translocation factor (TF) for Cd were calculated following [6] Abdal et al. (2021).

BCF = Conc. of metal in plant/Conc. of metal in solution

TF = Conc. of metal in shoot/Conc. of metal in root

The dry weight of metal-stressed plants was divided by the dry weights of non-stressed plants to get the value of TI [57] (Shabbir et al., 2020). 

### 4.9. Statistical Analyses

The data analysis was performed using one-way analysis of variance (ANOVA) with Statistix 8.1 software package. A least significant difference (LSD) test was used to compare the treatments at a significance level of 5% [58]. The PCA and Pearson’s correlation analysis was performed using XLSTAT 2014. 

## 5. Conclusions

The current study concluded that Cd and salinity in combination are more toxic for quinoa plants than their individual stress. The higher accumulation of Cd and Na caused the oxidative stress in quinoa tissues, resulting in the reduction in membrane stability, plant growth, and pigment contents. Plants were able to overcome the joint stress of salinity and Cd when SA and K were applied together. The increased resilience of quinoa against salinity and Cd was attributed to the reduced accumulation of Na and Cd by plants and increased activities of antioxidant enzymes. Thus, the combined application of SA and K effectively enhanced the ability of the quinoa plant to cope with the combined stress of salinity and Cd. However, the obtained results need further validation for salinity- and Cd-contaminated soils under field conditions.

## Figures and Tables

**Table 1:** Effect of salicylic acid (SA) and potassium (K) on growth attributes of quinoa grown under salinity (S), cadmium (Cd), and their combination.

Treatments	Shoot Length	Root Length	Shoot Dry Weight	Root Dry Weight
	(cm)	(cm)	(g plant ^−1^)	(g plant ^−1^)
Control	21 ± 0.5 a	19 ± 0.8 a	2.1 ± 0.08 a	0.70 ± 0.02 a
Cd	12 ± 0.4 d	11 ± 0.26 d	1.3 ± 0.04 d	0.38 ± 0.023 d
S	15 ± 0.5 b	13.5 ± 0.6 c	1.7 ± 0.05 b	0.48 ± 0.025 c
Cd-S	8.2 ± 0.5 e	8.0 ± 0.5 e	0.86 ± 0.07 e	0.23 ± 0.02 e
Cd-S-SA	12 ± 0.6 d	10 ± 0.3 d	1.3 ± 0.06 d	0.34 ± 0.015 d
Cd-S-K	14 ± 0.5 c	14 ± 0.6 c	1.5 ± 0.05 c	0.45 ± 0.02 c
Cd-S-SA-K	16.5 ± 0.6 b	16 ± 0.6 b	1.7 ± 0.04 b	0.53 ± 0.018 b

The presented data are the means of four replicates ± SE. Distinct letters within every column indicate a significant difference among the treatments at 5% probability level.

**Table 2:** Effect of salicylic acid (SA) and potassium (K) on physiological attributes of quinoa grown under salinity (S), cadmium (Cd), and their combination.

Treatments	Chl a	Chl b	Total Chl	Stomatal Conductance
	(µg g^−1^ FW)	(µg g^−1^ FW)	(µg g^−1^ FW)	(mmol m^−2^ s^−1^)
Control	460 ± 10 a	210 ± 5 a	670 ± 20 a	480 ± 10 a
Cd	320 ± 15 c	125 ± 8 d	445 ± 25 d	260 ± 20 d
S	400 ± 12 b	152 ± 5 c	552 ± 15 c	340 ± 14 c
Cd-S	230 ± 5 e	85 ± 6 e	315 ± 15 f	140 ± 18 f
Cd-S-SA	280 ± 15 d	127 ± 5 d	400 ± 22 e	215 ± 12 e
Cd-S-K	310 ± 8 c,d	142 ± 4 c	452 ± 20 d,e	280 ± 15 d
Cd-S-SA-K	400 ± 10 b	170 ± 6 b	590 ± 10 b	390 ± 10 b

The presented data are the mean of four replicates ± SE. Distinct letters within every column indicate a significant difference among the treatments at 5% probability level.

**Table 3:** Impact of SA and K on bioconcentration factor (BCF), translocation factor (TF), and tolerance index (TI) of quinoa under salinity (S), cadmium (Cd), and their combination with or without the addition of SA and K.

Treatment	BCF	TF	TI
Cd	3.74 ± 0.20 a	0.37 ± 0.04 b	62 ± 2.0 c
S	-	-	81 ± 3.0 b
Cd-S	3.56 ± 0.30 b	0.4 ± 0.02 a	41 ± 2.5 d
Cd-S-SA	3.11 ± 0.26 c	0.39 ± 0.01 a,b	62 ± 1.9 c
Cd-S-K	2.13 ± 0.15 d	0.33 ± 0.01 c	71 ± 2.0 b
Cd-S-SA-K	1.33 ± 0.17 e	0.27 ± 0.03 d	81 ± 2.1 a

The presented data are the mean of four replicates ± SE. Distinct letters within every column indicate a significant difference among the treatments at 5% probability level.

**Table 4:** Pearson correlation matrix of different attributes of quinoa under salinity and Cd treatments with or without supplementation of K and SA. Significant relationships between variables are indicated by values in bold.

Variables	SL	RL	RDW	SDW	TChl	Cond	SOD	CAT	POD	APX	H_2_O_2_	TBARS	MSI	RCd	SCd	SK	RK	SNa
RL	**0.980**																	
RDW	**0.996**	**0.987**																
SDW	**0.987**	**0.961**	**0.984**															
TChl	**0.971**	**0.955**	**0.973**	**0.974**														
Cond	**0.985**	**0.976**	**0.989**	**0.977**	**0.995**													
SOD	−0.139	−0.048	−0.169	−0.155	−0.139	−0.137												
CAT	−0.281	−0.173	−0.302	−0.321	−0.290	−0.277	**0.970**											
POD	−0.220	−0.091	−0.232	−0.234	−0.218	−0.210	**0.962**	**0.963**										
APX	−0.394	−0.289	−0.414	−0.416	−0.390	−0.385	**0.958**	**0.987**	**0.965**									
H_2_O_2_	**−0.909**	**−0.858**	**−0.918**	**−0.913**	**−0.944**	**−0.938**	0.403	0.529	0.496	0.621								
TBARS	**−0.978**	**−0.942**	**−0.981**	**−0.967**	**−0.975**	**−0.981**	0.296	0.426	0.380	0.534	**0.971**							
MSI	**0.965**	**0.950**	**0.973**	**0.975**	**0.991**	**0.989**	−0.188	−0.338	−0.262	−0.433	**−0.959**	**−0.975**						
RCd	**−0.797**	**−0.762**	**−0.793**	**−0.836**	**−0.819**	**−0.795**	0.305	0.465	0.315	0.507	0.740	**0.800**	**−0.784**					
SCd	**−0.841**	**−0.825**	**−0.839**	**−0.879**	**−0.869**	**−0.848**	0.198	0.365	0.208	0.416	**0.763**	**0.828**	**−0.838**	**0.987**				
SK	**0.924**	**0.975**	**0.942**	**0.882**	**0.901**	**0.933**	0.002	−0.080	−0.018	−0.207	**−0.803**	**−0.888**	**0.898**	−0.635	−0.712			
RK	**0.932**	**0.974**	**0.952**	**0.892**	**0.917**	**0.946**	−0.062	−0.145	−0.087	−0.271	**−0.844**	**−0.912**	**0.919**	−0.646	−0.718	**0.996**		
SNa	−0.596	−0.567	−0.617	−0.557	−0.566	−0.603	0.310	0.332	0.413	0.435	0.714	0.652	−0.638	0.101	0.146	−0.614	−0.653	
RNa	−0.614	−0.588	−0.635	−0.570	−0.590	−0.626	0.302	0.322	0.404	0.429	0.733	0.672	−0.657	0.120	0.166	−0.641	−0.680	**0.998**

## Data Availability

Data will be available as requested.
